# A guardian turned rogue: *TP53* promoter translocations rewire stress responses to oncogenic effectors in osteosarcoma

**DOI:** 10.1038/s41417-024-00749-9

**Published:** 2024-02-26

**Authors:** Nikolas Herold

**Affiliations:** 1 Childhood Cancer Research Unit, Department of Women’s and Children’s Health, Karolinska Institutet, Stockholm, Sweden; 2 Department of Paediatric Oncology, Astrid Lindgren Children’s Hospital, Karolinska University Hospital, Stockholm, Sweden

**Keywords:** Bone cancer, Cancer genetics

## Abstract

Osteosarcoma is the most prevalent malignant bone tumour in children, adolescents and young adults. Despite a multitude of aberrations present in osteosarcoma genomes, no recurrent driver mutations have been identified to date. In addition, unlike for other sarcoma entities, no functional fusion proteins resulting from chromosomal rearrangements have been reported. Part of the genetic complexity of osteosarcoma might, however, be explained by the association of osteosarcoma with germline and somatic mutations of the major tumour suppressor *TP53* that safeguards genomic integrity. By demonstrating that *TP53* promoter translocations resulting in transcriptionally active fusion genes are a recurrent event in osteosarcoma, long-learnt paradigms are challenged by a recent publication by Saba, Difilippo et al. Osteosarcoma no longer appears to be a fusion-negative tumour, and by hardwiring cellular stress responses that transactivate the *TP53* promoter to an oncogenic fusion partner, *TP53* can be subverted and turned into an oncogene.

## *TP53*—a tumour suppressor with oncogenic potential?

With a prevalence of ~40%, *TP53* encoding the stress-induced transcription factor p53 is the most frequently somatically altered gene in cancer [1]. *TP53* is generally considered a tumour suppressor and ”guardian of the genome” due to its ability to induce transcriptional programmes leading to cell-cycle arrest and either DNA repair or apoptosis [2]. *TP53*’s tumour suppressive properties may best be illustrated by the fact that germline mutations of *TP53* are associated with the cancer predisposition Li-Fraumeni syndrome that can give rise to a multitude of different malignancies, including osteosarcoma. However, discovered in 1979 in the context of virus-mediated malignant transformation [3], the first functional studies actually reported *TP53* to be an immortalising oncogene [4]. Later studies suggested that the observed oncogenic effects of *TP53* stemmed from the presence of non-synonymous point mutations [5]. This illustrates that the role of *TP53* in cancer is more complex as well as context-dependent and that not all somatic *TP53* alterations can be universally considered to inactivate a tumour suppressor. In particular, at least a subset of *TP53* missense mutations have been associated with oncogenic functions in animal experiments, and this has led to a hypothesis that certain *TP53* mutations might be considered ”separation-of-function” mutations altering the balance of pro- and antiproliferative effects (summarised in [6]).

## *TP53* promoter translocation as a separation-of-function paradigm in osteosarcoma

A recent genetic and transcriptomic study on 148 osteosarcoma patients published by Saba, Difilippo et al. [7] provides a novel paradigm in which *TP53* rearrangements can simultaneously result in inactivation of p53 tumour suppressor functions and activation of oncogenic pathways by fusing the *TP53* promoter region to new target genes (promoter swapping). In ~40% of analysed cases, evidence for *TP53* promoter translocation was found, and in ~20% of cases, a putative fusion partner was readily identified. The authors further demonstrated that the resulting fusions were in-frame, and transcription of the *TP53* fusion partner was increased. At the same time, expression of *TP53* was lost, suggesting that promoter translocations of one *TP53* allele co-occur with inactivating genetic aberrations of the other allele in osteosarcoma, and a selective advantage can be inferred. Functionally, the authors elegantly showed that DNA damage induced by cisplatin (a front-line drug to treat osteosarcoma) readily induced expression of the fusion partner in several different cell lines with different fusion partners.

**Fig. 1:**
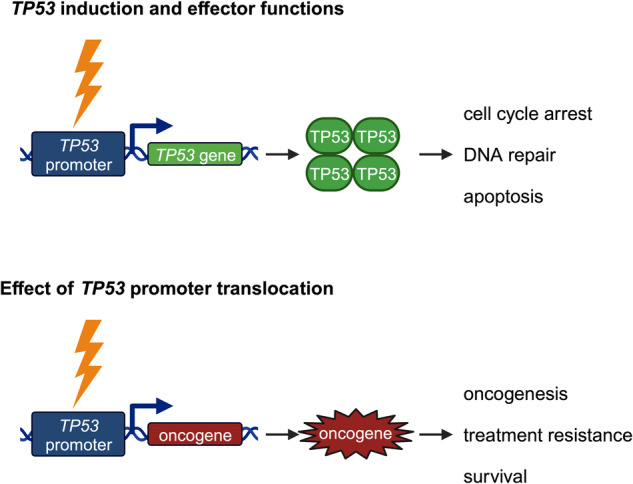
Oncogenic effects *TP53* promoter translocation. Upper panel: Cellular stress results in transactivation of the *TP53* promoter, leading to transcription and eventually translation of TP53 protein whose transcriptional activity is responsible for physiological stress responses like cell cycle arrest, DNA repair and apoptosis. Lower panel: Translocation of the *TP53* promoter brings an oncogene under the control of cellular stress, which can cause oncogenesis, treatment resistance and tumour survival.

Hence, these translocations do not only bring potential oncogenes under the control of the *TP53* promoter, but they also disrupt the expression of a functional p53 protein. In essence, this results in both disruption of safeguarding *TP53* responses upon, e.g., replication stress, reactive oxygen species and DNA damage and rewiring of the upstream stress response machinery to effector functions of a potentially oncogenic fusion partner (Fig. 1). In fact, a subset of the identified fusion partners had already been implicated in the pathobiology of osteosarcoma and other malignancies.

Of note, this separation-of-function paradigm (a term originally developed for specific TP53 missense mutations [6]) combines a canonical loss-of-function of the *TP53* gene body with the overexpression of an oncogene through *TP53* promoter hijacking as described in other tumours like lipoblastoma [8].

## Osteosarcoma—a fusion-driven cancer?

Interestingly, *TP53* hotspot mutations and *TP53* promoter translocation were mutually exclusive even though the global gene expression patterns were similar. Furthermore, *TP53* promoter translocations positively correlated with young age and a number of chromosomal rearrangements.

The high prevalence of these fusions in osteosarcoma, in particular in young patients, suggests a distinct role in the oncogenesis of osteosarcoma. This is of fundamental importance as osteosarcoma has hitherto not been considered a fusion-driven cancer, and fusions that arise by chance in the highly rearranged osteosarcoma genome have been reported to be out-of-frame and, thus, non-functional [9]. More experimental evidence, however, is required to answer the question of whether *TP53* promoter translocations are indeed an early oncogenic event and whether this is causally linked to the subsequent accumulation of structural variants. Recently, single-cell analyses in a mouse model of pancreatic ductal adenocarcinoma showed that loss-of-function mutations in *TP53* precede extensive copy number variations [10]. A similar approach is conceivable also for osteosarcoma research. Alternatively, spatiotemporal sampling and genetic analyses followed by clonal deconvolution could be considered in primary patient material as demonstrated for other diagnoses [11].

## Conclusions

Identification of recurrent *TP53* promoter translocations resulting in functional fusion genes is a major new paradigm for osteosarcoma in particular and for cancer research in general.

Future studies should be directed at exploring whether the subgroup of *TP53*-fusion-positive osteosarcoma comprises a distinct clinicobiological subset of osteosarcoma with respect to metastatic potential, therapeutic responses and immunological control.
